# Sample Processing Impacts the Viability and Cultivability of the Sponge Microbiome

**DOI:** 10.3389/fmicb.2016.00499

**Published:** 2016-04-12

**Authors:** Ana I. S. Esteves, Nimra Amer, Mary Nguyen, Torsten Thomas

**Affiliations:** Centre for Marine Bio-Innovation, School of Biological, Earth and Environmental Sciences, The University of New South WalesKensington, NSW, Australia

**Keywords:** microbial cultivation, sponge microbiome, bacterial viability, microbial diversity, cultivation bias

## Abstract

Sponges host complex microbial communities of recognized ecological and biotechnological importance. Extensive cultivation efforts have been made to isolate sponge bacteria, but most still elude cultivation. To identify the bottlenecks of sponge bacterial cultivation, we combined high-throughput 16S rRNA gene sequencing with a variety of cultivation media and incubation conditions. We aimed to determine the extent to which sample processing and cultivation conditions can impact bacterial viability and recovery in culture. We isolated 325 sponge bacteria from six specimens of *Cymbastela concentrica* and three specimens of *Scopalina* sp. These isolates were distributed over 37 different genera and 47 operational taxonomic units (defined at 97% 16S rRNA gene sequence identity). The cultivable bacterial community was highly specific to its sponge host and different media compositions yielded distinct microbial isolates. Around 97% of the isolates could be detected in the original sponge and represented a large but highly variable proportion (0.5–92% total abundance, depending on sponge species) of viable bacteria obtained after sample processing, as determined by propidium monoazide selective DNA modification of compromised cells. Our results show that the most abundant viable bacteria are also the most predominant groups found in cultivation, reflecting, to some extent, the relative abundances of the viable bacterial community, rather than the overall community estimated by direct molecular approaches. Cultivation is therefore shaped not only by the growth conditions provided, but also by the different cell viabilities of the bacteria that constitute the cultivation inoculum. These observations highlight the need to perform experiments to assess each method of sample processing for its accurate representation of the actual *in situ* bacterial community and its yield of viable cells.

## Introduction

Culturing is an important tool to study the physiology and ecological function of microorganisms (Stewart, 2012) and it can provide access to bacteria that have eluded detection with molecular approaches (Donachie et al., 2007; Shade et al., 2012). Culturing, however, comes with its downfalls, as many bacteria that are dominant in certain ecosystems remain recalcitrant to cultivation (Joint et al., 2010). To successfully cultivate a given microorganism, it is important that all its physiological and metabolic needs are met (Leadbetter, 2003). Therefore, several innovative culturing approaches have been developed, including complex microfluidic and laser manipulation systems (Fröhlich and König, 2000; Zhang and Liu, 2008; Liu et al., 2009; Yamaguchi et al., 2009), high-throughput cultivation technologies based on dilution-to-extinction and microencapsulation (Connon and Giovannoni, 2002; Zengler et al., 2005; Ben-Dov et al., 2009), diffusion devices that allow the exchange of small molecules with the environment (Kaeberlein et al., 2002; Bollmann et al., 2007; Nichols et al., 2010), filters and membrane systems to simulate the natural environment (de Bruyn et al., 1990; Ferrari et al., 2008), co-culture approaches (Ohno et al., 2000; Nichols et al., 2008), formulation of new media compositions based on metagenomic information (Tyson et al., 2005) and establishment of growth conditions to mimic the natural environment with low-nutrient media and longer incubation times (Song et al., 2009). Collectively, these approaches have made significant breakthroughs by increasing the diversity and recovery rates of bacteria retrieved in culture and enabling the cultivation of ecologically relevant microorganisms, like the SAR11 clade (Rappé et al., 2002; Bollmann et al., 2007).

Sponges (phylum Porifera) harbor complex microbial communities with recognized ecological and biotechnological interest (Hentschel et al., 2006; Taylor et al., 2007). Several studies have reported a range of bioactivities from sponges, including antitumoral (Simmons et al., 2005), antiviral (Esteves et al., 2011), and antimicrobial (Newbold et al., 1999) activities, and in some cases their production could be attributed to bacteria rather than the host (Waters et al., 2014; Wilson et al., 2014). These observations, in conjunction with the fact that the sponge holobiont arguably represents the oldest extant association between prokaryotes and metazoans (Peterson and Butterfield, 2005), render sponges an important model system. While in the past decade a comprehensive picture of the phylogenetic diversity and composition of the sponge microbiome has been generated through culture-independent methods (Webster and Taylor, 2012), the *in situ* activity and function of these microbial symbionts are now the major research focus (Thomas et al., 2010; Hentschel et al., 2012; Radax et al., 2012; Ribes et al., 2012; Fan et al., 2013).

Extensive cultivation efforts have made use of several alternative techniques to isolate sponge-associated bacteria, including the use of low-nutrient and seawater-based media, the addition of host tissue and/or aqueous and organic sponge extracts, the use of alternative carbon sources (chitin, mucin, casein, aminoacids, urea, simple and complex sugars), the addition of antioxidant molecules (catalase and sodium pyruvate), the addition of antibiotics to inhibit fast growing bacteria, the incubation at different oxygen regimes and for longer times (Santavy et al., 1990; Olson et al., 2000; Hentschel et al., 2001; Webster et al., 2001; Sfanos et al., 2005; Lavy et al., 2014), and use of diffusion devices (Jung et al., 2014; Steinert et al., 2014) and floating filters (Sipkema et al., 2011). These different approaches resulted in increased diversity of sponge isolates yielding previously uncultured bacteria and improved cultivability rates in some cases up to 14% (Sipkema et al., 2011). However, discrepancies were found between community composition defined through cultivation-dependent and -independent approaches. For example, cultivable bacterial counts were 2–3 orders of magnitude lower than direct bacterial cell counts in the sponge and there was little to no overlap between cultured isolates and uncultured community data (Friedrich et al., 2001; Webster et al., 2001; Li et al., 2011; Hardoim et al., 2014; Montalvo et al., 2014). Few studies have addressed these disparities in detail to understand what factors impact or limit cultivation.

The present study was designed to identify the bottlenecks of bacterial cultivation by evaluating the extent to which sample processing and cultivation conditions can impact bacterial viability and recovery. Two model sponges, *Cymbastela concentrica* and *Scopalina* sp., were chosen for this purpose, as their microbial communities have been well characterized by 16S rRNA gene sequencing and fingerprinting (Taylor et al., 2004, 2005; Thomas et al., 2010), metagenomics (Fan et al., 2012b) and metaproteomics (Liu et al., 2012) and as they have also shown biotechnological potential for secondary metabolite production (Penesyan et al., 2011; Woodhouse et al., 2013). However, the cultivability of bacterial symbionts present in these two sponges has not been explored. We therefore combined high-throughput 16S rRNA gene sequencing with a variety of cultivation media and incubation conditions to determine the relative abundance and taxonomic affiliation of bacteria that remained viable after microbial cell enrichment and were amenable to cultivation.

## Materials and Methods

### Sponge Collection and Sample Preparation

Sampling took place at Bare Island in Botany Bay, NSW, Australia (33° 59′S, 151° 14′E) in three independent campaigns. *C. concentrica* samples were collected on 26-09-2013 (samples Cymb1-3) and 21-02-2014 (samples Cymb4-6); *Scopalina* sp. samples were collected on 12-12-2013 (samples Scop1-3). On each sampling event, three sponge specimens were collected by SCUBA diving at depths of 7–10 m and placed individually with surrounding seawater into Ziploc^®^ bags. Samples were transported to the laboratory (within 30 min) in a cooling box and immediately processed as described below. Sponge species were identified based on their distinct macroscopic morphologies and spicule observation using optical microscopy and in comparison to previous molecular identification (Fan et al., 2012b).

All work henceforth was performed in a laminar flow cabinet under sterile conditions. Sponges were cleaned of any debris and/or foreign macroscopic material and rinsed with sterile natural seawater (NSW). Cleaned sponges were then cut into 0.5 cm^3^ pieces and washed four times with sterile calcium magnesium-free sea water (CMFSW; 25 g NaCl, 0.8 g KCl, 1 g Na_2_SO_4_ and 0.04 g NaHCO_3_ per 1 L) on a platform rocker at maximum speed, for 10 min at 4°C, to remove loosely attached cells. After washing, one sponge piece was immediately stored at -80°C for DNA extraction and 36 pieces were kept on ice for direct inoculation of agar plates. Around 2 g was further processed for microbial cell enrichment and the remaining was used for sponge extract preparation (see next sections).

### Microbial Cell Enrichment

Microbial cells were enriched from sponge tissue according to the method described by Thomas et al. (2010) with minor modifications. Briefly, the washed sponge material was cut into 0.5 cm^3^ cubes and homogenized on ice for 10–30 s in 50 mL of CMFSW, using a dispersing homogenizer (Ultra-Turrax TR50, IKA). The homogenate was then filtered through a 125 μm metal sieve and the filtrate was centrifuged for 15 min at 100 × *g* and 4°C to remove remaining sponge cells and tissue debris. The supernatant was centrifuged twice for 15 min at 300 × *g* and 4°C and afterward filtered twice through an 11 μm filter using a vacuum filtration unit. An additional filtration step through a 3 μm filter was performed, which has been previously optimized for the removal of eukaryotic cells and organelles whilst avoiding removal of microorganisms (Fan et al., 2012b). The final filtrate was centrifuged for 15 min at 15000 × *g* and 4°C to pellet microbial cells, washed three times with CMFSW and finally resuspended in 10 mL of CMFSW.

Three 1 mL aliquots of the final microbial cell suspension were immediately taken: one was used for the preparation of dilutions for inoculation and another was stored at -80°C for community DNA extraction. The third aliquot was treated with propidium monoazide dye (PMA; Biotium Hayward, CA, USA) according to the manufacturer’s instructions. PMA is a cell membrane-impermeable dye and inhibits PCR amplification by permanent DNA modification. It can thus be used to discriminate bacteria by selective modification of DNA from dead cells with compromised membrane integrity, while leaving DNA from viable cells intact. After photo-activation of the dye using a 750 W halogen lamp, cells were pelleted and stored at -80°C for subsequent DNA extraction. The remaining cell suspension was stored at -80°C in 20% (v/v) glycerol/NSW solution until further use.

### Sponge Extract Preparation

Sponge specimens belonging to the same species were pooled together and homogenized in 10 mL of sterile NSW per gram of sponge sample, using a food processor (Tongtel mini-chopper KA0201) at maximum speed on ice until complete tissue disruption. This homogenate was then incubated for 30 min, on ice, with stirring, filtered through a 125 μm metal sieve and centrifuged at 15000 × *g* for 30 min at 4°C. The supernatant was filtered through a series of vacuum filtration steps, using decreasing pore sizes (12, 3, and 0.2 μm). This sponge extract was incorporated in some of the cultivation media and the rest was stored at -80°C until further use.

### Isolation of Sponge Associated Bacteria

Culture media were either newly formulated or adapted from references to mimic physiological conditions inside the sponge and target specific bacterial taxa previously found in these sponges (Fan et al., 2012b). Low nutrient media, formulated using diluted marine broth or sterile natural seawater, account for the nutrient limitation that bacteria might encounter under high cell densities. Silica is part of the spicules that constitute the sponge skeleton along with spongin and collagenous fibers, which might provide important inorganic or organic nutrients. Sponge extract was filter sterilized and added to the respective media after autoclaving to account for sponge-specific nutrients and/or infochemical requirements. Functions related to anaerobic metabolism (e.g., denitrification) and ammonium oxidation were previously found to be significantly enriched in these sponges (Fan et al., 2012b). Anaerobic incubations with NO_3_^-^ supplemented media and aerobic incubations with NH_4_^+^ amended media were performed to account for denitrification and ammonium oxidation, respectively.

A total of 10 agar-based media, one gellan-gum medium and five liquid media were used, spanning different nutrient concentrations, oxygenation conditions and growth matrix (**Table 1**). Filter-sterilized cycloheximide (20 mg/L) and nystatin (25 mg/L) were added to the media after autoclaving to inhibit fungal growth, and nalidixic acid (20 mg/L) was used to inhibit fast-growing gram-negative bacteria that would otherwise overgrow and prevent isolation of slow-growing bacteria (Webster et al., 2001).

**Table 1:** Composition of cultivation media and incubation conditions used in this study for the isolation of sponge bacteria.

Code	Composition	Target	O_2_ conditions	Growth matrix	Reference
MB1/2	18.7 g Marine Broth 2216 (BD Difco), 500 mL MilliQ^®^ water, 500 mL NSW^a^, 15 g Agar Noble (BD Difco)	Generalist bacteria	Aerobic	Agar plates	This study
MB20	1.87 g Marine Broth 2216, 25 g SiO_2_ (0.5–10 μm, Sigma-Aldrich), 1 L NSW, 15 g Agar Noble	Low nutrient bacteria	Aerobic	Agar plates	This study
MB20SE	1.87 g Marine Broth 2216, 20 mL sponge extract, 25 g SiO_2_, 980 mL NSW, 15 g Agar Noble	Low nutrient bacteria	Aerobic	Agar plates	This study
NSW	25 g SiO_2_, 1 L NSW, 15 g Agar Noble	Low nutrient bacteria	Aerobic	Agar plates	This study
NSWSE	25 g SiO_2_, 100 mL sponge extract, 900 mL NSW, 15 g Agar Noble	Low nutrient bacteria	Aerobic	Agar plates	This study
ASP	25 g SiO_2_, 100 mL sponge extract, 900 mL CMFSW, 8 g gellan gum (Gelzan^TM^ CM, Sigma-Aldrich)^b^	Spongin binding bacteria	Aerobic	Gellan gum plates	This study
ASWNH4	10 mg (NH_4_)_2_SO_4_, 1 L ASW^c^, 15 g Agar Noble	Nitrifying marine bacteria	Aerobic	Agar plates	Adapted from McCaig et al. (1994)
MB20NO3	1.87 g Marine Broth 2216, 2 g KNO_3_, 1 L NSW, 15 g Agar Noble, pH = 7.5	Denitrifying marine bacteria	Anaerobic	Agar plates	Adapted from Brettar et al. (2001)
4C	69 mg NaNO_2_, 100 mg MgSO_4_.7H_2_O, 6 mg CaCl_2_.2H_2_O, 1.7 mg K_2_HPO_4_, 100 μL of micronutrients solution^d^, 700 mL NSW, 300 mL MilliQ^®^ water, 15 g Agar Noble	NO_2_^-^ oxidisers	Aerobic	Agar plates	Adapted from Watson and Waterbury (1971)
ASW	1 g glucose, 1 L ASW, 15 g Agar Noble	N_2_ fixers	Aerobic and anaerobic	Agar plates	Glucose nitrogen-free salt solution adapted from Atlas (2005)
R2A	18.2 g R2A Agar (BD Difco), 750 mL NSW, 250 mL MilliQ^®^ water	Gammaproteobacteria (Oceanospirilaceae)	Aerobic	Agar plates	Adapted from Suzuki et al. (1997)
SNAX	25.5 mg K_2_HPO_4_, 90 mg KNO_3_, 1 mg Na_2_CO_3_, 0.5 mg Na_2_ EDTA.2H_2_O, 13 mg (NH_4_)_2_SO_4_, 100 μL micronutrients solution^d^, 750 mL NSW, 250 mL MilliQ^®^ water	Cyanobacteria	Aerobic	Liquid	Adapted from Waterbury et al. (1986)
NSWFF	25 g SiO_2_, 1 L NSW	Low nutrient bacteria sensitive to agar	Aerobic	Floating filters	Adapted from Sipkema et al. (2011)
NSWSEFF	25 g SiO_2_, 100 mL sponge extract, 900 mL NSW	Low nutrient bacteria sensitive to agar	Aerobic	Floating filters	Adapted from Sipkema et al. (2011)
MB20FF	1.87 g Marine Broth 2216, 25 g SiO_2_, 1 L NSW	Low nutrient bacteria sensitive to agar	Aerobic	Floating filters	Adapted from Sipkema et al. (2011)
MB20SEFF	1.87 g Marine Broth 2216, 20 mL sponge extract, 25 g SiO_2_, 980 mL NSW	Low nutrient bacteria sensitive to agar	Aerobic	Floating filters	Adapted from Sipkema et al. (2011)

*^a^NSW, Natural seawater; ^b^Spongin slices were obtained from a commercial natural sponge cut thinly into 2–3 mm thick slices with a scalpel and autoclaved in CMFSW. Excess CMFSW was then removed with sterile paper filters and slices were used to cover the bottom of Petri dishes and overlaid with the gellan gum mixture; ^c^ASW (artificial sea water) composition: 23.38 g/L NaCl, 2.41 g/L MgSO_4_, 1.19 g/L MgCl_2_, 1.47 g/L CaCl_2_.2H_2_O, 0.75 g/L KCl, 0.17 g/L NaHCO_3_; ^d^Micronutrients solution composition: 6 mg/mL iron citrate, 1.4 mg/mL MnCl_2_.4H_2_O, 20 μg CoCl_2_.6H_2_O, and 0.22 mg ZnSO_4_.7H_2_O*.

The microbial cell suspension was serially diluted to 10^-5^ in CMFSW. One hundred microliters of dilutions 10^-3^ to 10^-5^ were spread in triplicates onto agar plates corresponding to media NSW, NSWSE, MB20, MB20SE, MB1/2 and R2A. For media ASWNH4, 4C, ASW and MB20NO3, only dilution 10^-3^ was used and for media ASP, dilutions 10^-1^ and 10^-3^ were plated. For cyanobacteria cultivation, 100 mL of SNAX media was directly inoculated with one piece of washed sponge using sterile tweezers. One piece of sponge was also directly placed onto all solid media, in triplicates, using sterile tweezers.

For floating filter cultivation, the methodology described in Sipkema et al. (2011) was followed with minor modifications. Briefly, white polycarbonate filters (Nuclepore Track-etch membrane, Whatman) with 47 mm diameter and 0.2 μm pore size were autoclaved and mounted on a sterile glass filter holder. Filters were first rinsed with 10 mL CMFSW before 10 mL of diluted cell suspension was filtered. Two different inoculum concentrations (10^-3^ and 10^-4^) were used in triplicate for four liquid media (**Table 1**). Filters were then gently placed on top of the liquid medium in a small Petri dish (Ø60 mm × 15 mm) using sterile tweezers.

To create anaerobic conditions, Petri dishes were inserted in 3.5 L anaerobic jars (Oxoid, Thermo Scientific) together with anaerobic gas generating sachets (AnaeroGen 3.5L Sachet, Oxoid, Thermo Scientific) and a colorimetric indicator strip (Oxoid^TM^ Resazurin Anaerobic Indicator, Thermo Scientific) to monitor oxygen levels, and quickly sealed.

To assess the influence of freezing on the cultivability of the microbial cell suspension, after 1 month, one aliquot stored in glycerol was slowly thawed on ice, diluted to 10^-2^ and 100 μL were spread on MB1/2 medium in triplicates. These samples will be referred to as MBdef.

Plates (solid media and floating filters) were incubated in the dark at 18°C for 6 months with monthly visual inspection. Colonies with different morphologies were purified by successive restreaking on MB1/2 plates. Isolate colonies were transferred to 3 mL of sterile half strength liquid marine broth (18.7 g Difco Marine Broth 2216, 500 mL NSW, 500 mL MilliQ^®^ water) and incubated at 18°C, with 200 rpm shaking, until a dense culture was formed (3–5 days). An aliquot of each culture was stored at -80°C in 20% glycerol until further use. Two milliliters of the remaining culture were centrifuged at 15000 *g* for 10 min and the resulting bacterial pellet was stored at -20°C for DNA extraction.

For cyanobacteria isolation, 100 mL of SNAX media was poured into sterile conical flasks and incubated at room temperature (24–26°C), with shaking and under continuous illumination with cool white light (∼20 μE m^-2^s^-1^). Cyanobacteria growth was observed after 3 months incubation. Cultures were then transferred to 24 well culture plates (Corning Cellbind, Sigma-Aldrich) in SNAX media (without supplemented antibiotics) and incubated for 3–4 weeks at room temperature, static and with continuous illumination (∼15–20 μE m^-2^ s^-1^). Cyanobacteria filaments or colonies were picked using a sterile pipette, serially diluted and sub-cultured into new 24 well plates. This was repeated until pure cultures were observed under the microscope. Pure cultures were transferred and maintained in cell culture flasks (T25, T175 Corning cell culture flask, Sigma-Aldrich) at room temperature, static and under continuous illumination (∼15–20 μE m^-2^ s^-1^).

### Isolate DNA Extraction and Sequencing

DNA from bacterial isolates was extracted from previously stored pellets using the DNeasy Blood and Tissue extraction kit (Qiagen), according to the manufacturer’s instructions. 16S rRNA gene fragments were amplified using the bacterial universal primers F27 (5′-AGA GTT TGA TCM TGG CTC AG-3′) and R1492 (5′-TAC GGY TAC CTT GTT ACG ACT T-3′; Weisburg et al., 1991) and the cyanobacteria-specific reverse primer 809R (5′-GCT TCG GCA CGG CTC GGG TCG ATA-3′; Al-Tebrineh et al., 2010), yielding fragments of approximately 1500 and 800 bp in length, respectively. PCR amplification reaction mixtures (25 μL) consisted of 12.5 μL EconoTaq Plus Green 2x Master Mix (Lucigen Corporation, Middleton, WI, USA), 0.5 μL of each primer (10 μM) and 1 μL template DNA. Thermal cycling started with 5 min at 94°C for the initial denaturation step, 30 cycles of 94°C for 30 s, 56°C for 30 s and 72°C for 90 s, ending with a final extension step of 10 min at 72°C (Esteves et al., 2013).

Amplicons were visually inspected for size and success of amplification with a Gel documentation system (GelDoc, Bio-Rad) after electrophoresis in a 1% agarose gel stained with 30 ppm of GelRed^TM^ 10000x solution in DMSO (Biotium, Hayward, CA, USA). PCR products were then cleaned with Sephadex G50 (DNA grade, fine, Sigma Aldrich) columns and 20–50 ng were used in the sequencing reaction with the BigDye Terminator v3.1 (Applied Biosystems, Austin, TX, USA), according to the protocols provided by the sequencing facilities. Sequencing reactions were purified using Sephadex G50 columns and sequenced using the forward primer only in an Applied Biosystems 3730 DNA Analyzer, at the Ramaciotti Centre for Genomics (University of New South Wales, Sydney, NSW, Australia). For forward 16S rRNA gene sequences smaller than 800 bp, sequencing was also performed using the reverse primer. Sequences were manually trimmed using Sequence Scanner v1.0 software (Applied Biosystems) and assembled (when applicable) using DNA Baser v3.5.0 (Heracle BioSoft). All isolate 16S rRNA gene sequences were deposited in the EMBL Nucleotide Sequence Database^1^ under accession numbers LN878321-LN878646.

### Isolate 16S rRNA Gene Sequence Analysis

Bacterial isolates were taxonomically classified up to genus level based on the 16S rRNA gene sequences using the Classifier tool of the Ribosomal Database Project release 11 (Wang et al., 2007) with an 80% confidence threshold. Uncorrected pairwise evolutionary distances were calculated using the algorithms implemented in the MOTHUR software package v1.33.3 (Schloss et al., 2009). Sequences were clustered into OTUs at 100% (unique sequences) and 97% identity, using the furthest neighbor algorithm. These OTUs, resulting from the independent cluster analysis of isolate sequences only, will be referred to as ‘iOTUs’ throughout the manuscript.

To determine whether the collection of cultivated bacteria derived from the two different sponge species were significantly different in their composition, library shuﬄing analysis of the size normalized sequence libraries was performed using the tool ‘libshuff’ as implemented by MOTHUR. Bacterial richness and diversity estimators (Chao1 and inverted Simpson, respectively) were also calculated for each sponge species after size normalization (*n* = 137 sequences per sponge species).

### Community DNA Extraction and 16S rRNA Gene Sequencing

For each collected sponge specimen, three different samples were analyzed: the unprocessed sponge tissue and the microbial cell pellet, before and after PMA treatment. These will be hereafter referred to as sponge (SP), bacterial pellet (BAC), and viable fraction (PMA), respectively. For SP samples, DNA was directly extracted from a 0.5 cm^3^ piece of sponge. For BAC samples, DNA was extracted from a 1 mL aliquot of the microbial pellet, prepared as previously described in Section “Microbial Cell Enrichment.” For PMA samples, a 1 mL aliquot of the microbial pellet was treated with PMA dye, as described in Section “Microbial Cell Enrichment,” cells were pelleted by centrifugation at 15000 × *g* for 10 min and this pellet was then used for DNA extraction.

Total community DNA was extracted using the PowerSoil^®^ -htp 96 well DNA isolation kit (Mo Bio, Inc., Carlsbad, CA, USA) according to the manufacturer’s protocols. DNA extracts were visually checked for quality by agarose gel electrophoresis and quantified spectrophotometrically (NanoDrop ND-1000). The conserved regions flanking the v4 region of the 16S rRNA gene found in bacteria and archaea were targeted using the primers 515F (5′-GTG CCA GCM GCC GCG GTA A-3′) and 806R (5′-GGA CTA CHV GGG TWT CTA AT-3′). PCR amplification of the 16S rRNA genes and Illumina MiSeq amplicon sequencing were performed at the Ramaciotti Centre for Genomics (University of New South Wales, Sydney, NSW, Australia), according to the methodology described by Caporaso et al. (2012). Raw Illumina reads were deposited in the Sequence Read Archive^2^ under Bioproject nr PRJNA292036.

### Combined Community and Isolate v4-16S rRNA Gene Sequence Processing and Analysis

Raw forward and reverse sequence reads (150 bp) from community samples were assembled into contigs, quality filtered, taxonomically classified and clustered into operational taxonomic units (OTUs) using MOTHUR (v1.33.3, Schloss et al., 2009). All sequences with ambiguities, more than eight homopolymers and sequence length greater than 300 bp were discarded. Filtered amplicon sequences and isolate sequences were pooled, aligned to the Silva 16S rRNA gene reference alignment v102 (Quast et al., 2013), screened to include only overlapping regions, pre-clustered to merge all sequences within two mismatches (differences = 2) and checked for chimeras using the UCHIME algorithm (Edgar et al., 2011).

Because RDP reference taxonomy does not separate chloroplasts from cyanobacteria, sequences were first classified using the Silva reference taxonomy v119 (Quast et al., 2013) with a 60% confidence threshold. All sequences classified as chloroplasts were removed and the remaining sequences were then re-classified using the RDP training set release 9 (Cole et al., 2014), discarding all sequences classified as unknown or mitochondria. The quality-filtered sequences were then clustered into OTUs at 97% similarity. These OTUs, resulting from the sequence cluster analysis of uncultured microorganisms, will be referred to as ‘uOTUs’ throughout the manuscript. All rare uOTUs (*n* ≤ 2) were removed from further analysis. Sequence counts for cultivation independent community data were normalized for gene copy number using Copyrighter v0.46 (Angly et al., 2014) and the Greengenes classification version 13/05 (DeSantis et al., 2006) and afterward sub-sampled to the size of the smallest sample.

Data analysis was carried out using both full and size normalized OTU tables. The full OTU tables were used to determine the absolute number and identity of specific and shared OTUs in each sample. The normalized data was used in the quantitative comparison of bacterial richness, diversity, and community structure between samples.

To confirm taxonomic affiliation, an optimal maximum-likelihood tree was generated to include all 16S rRNA gene sequences from isolates, representative sequences for corresponding uOTUs and closest sequences in the SILVA database (Quast et al., 2013). The resulting phylogenetic tree can be found in the Supplementary Information (Supplementary Figure S2).

## Results

### Composition of the Cultivated Fraction

A total of 325 bacteria were isolated from nine sponge specimens: 149 isolates from six specimens of *C. concentrica* and 176 isolates from three specimens of *Scopalina* sp., with an average of 25 ± 6 (standard deviation) and 59 ± 16 bacterial isolates per specimen for *C. concentrica* and *Scopalina* sp., respectively. 16S rRNA gene sequences were obtained for 311 bacterial isolates, with sizes ranging from 670 to 1429 bp. Bacteria were distributed among 37 different genera and sequences clustered into 47 iOTUs at 97% similarity (**Table 2**). Although, a higher number of isolates and higher counts of bacteria per sponge specimen was retrieved from *Scopalina* sp., *C. concentrica* harbors a slightly richer cultivable community, spanning 25 bacterial genera and 32 iOTUs compared to 19 taxa and 19 iOTUs found in *Scopalina* sp. However, this difference is not statistically significant (one-way ANOVA).

**Table 2:** Phylogenetic classification and relative abundances (RA) of bacteria isolated from *C. concentrica* (Cymb) and *Scopalina* sp. (Scop); in brackets: number of 16S rRNA gene sequences, number of OTU clusters formed at 97% sequence similarity.

Phylum	Class	Order	Family	Genus	Cymb	Scop	Total
*Proteobacteria*	*Alpha*	*Rhodobacterales*	*Rhodobacteraceae*	*Pseudovibrio*	54.4 (74;1)	39.4 (69;1)	46.0 (143;1)
				*Ruegeria*	2.9 (4;1)	29.1 (51;1)	17.7 (55;1)
				*Labrenzia*	2.2 (3;1)	21.7 (38;1)	13.2 (41;1)
				*Paracoccus*	0	0.6 (1;1)	0.3 (1;1)
				Unclassified	2.2 (3;3)	0.6 (1;1)	1.3 (4;4)
		*Sphingomonadales*	*Sphingomonadaceae*	*Sphingopyxis*	0	0.6 (1;1)	0.3 (1;1)
		*Kiloniellales*	*Kiloniellaceae*	*Kiloniella*	0.7 (1;1)	0	0.3 (1;1)
	*Gamma*	*Vibrionales*	*Vibrionaceae*	*Vibrio*	8.8 (12;2)	0.6 (1;1)	4.2 (13;3)
				*Enterovibrio*	4.4 (6;1)	0	2.0 (6;1)
				*Photobacterium*	1.5 (2;1)	0	0.6 (2;1)
				Unclassified	2.2 (3;1)	0	1.0 (3;1)
		*Pseudomonadales*	*Moraxellaceae*	*Acinetobacter*	2.2 (3;1)	0	1.0 (3;1)
		*Oceanospirillales*	*Oceanospirillaceae*	*Neptuniibacter*	0.7 (1;1)	0	0.3 (1;1)
		*Xanthomonadales*	*Xanthomonadaceae*	*Luteimonas*	0	0.6 (1;1)	0.3 (1;1)
		*Alteromonadales*	*Colwelliaceae*	*Colwellia*	0.7 (1;1)	0	0.3 (1;1)
			*Alteromonadaceae*	*Marinobacter*	0.7 (1;1)	0	0.3 (1;1)
			*Shewanellaceae*	*Shewanella*	0	0.6 (1;1)	0.3 (1;1)
			*Pseudoalteromonadaceae*	*Pseudoalteromonas*	0.7 (1;1)	0	0.3 (1;1)
	*Delta*	*Desulfovibrionales*	*Desulfovibrionaceae*	*Desulfovibrio*	1.5 (2;1)	0	0.6 (2;1)
*Firmicutes*	*Clostridia*	*Clostridiales*	*Peptostreptococcaceae*	*Tepidibacter*	0.7 (1;1)	0	0.3 (1;1)
	*Bacilli*	*Bacillales*	*Bacillaceae 1*	*Bacillus*	6.6 (9;5)	0.6 (1;1)	3.2 (10;5)
			*Bacillaceae 2*	*Oceanobacillus*	0.7 (1;1)	0	0.3 (1;1)
				*Virgibacillus*	0	0.6 (1;1)	0.3 (1;1)
			*Bacillales_Incertae Sedis XII*	*Exiguobacterium*	0	0.6 (1;1)	0.3 (1;1)
			*Planococcaceae*	*Paenisporosarcina*	0	0.6 (1;1)	0.3 (1;1)
*Bacteroidetes*	*Flavobacteriia*	*Flavobacteriales*	*Flavobacteriaceae*	*Tenacibaculum*	1.5 (2;1)	1.1 (2;1)	1.3 (4;2)
				*Croceibacter*	0	0.6 (1;1)	0.3 (1;1)
				*Maribacter*	0.7 (1;1)	0	0.3 (1;1)
*Actinobacteria*	*Actinobacteria*	*Actinomycetales*	*Micrococcaceae*	*Micrococcus*	0	0.6 (1;1)	0.3 (1;1)
			*Dermabacteraceae*	*Brachybacterium*	0	0.6 (1;1)	0.3 (1;1)
			*Microbacteriaceae*	*Microbacterium*	0	0.6 (1;1)	0.3 (1;1)
			*Dietziaceae*	*Dietzia*	0	0.6 (1;1)	0.3 (1;1)
			*Streptomycetaceae*	*Streptomyces*	0.7 (1;1)	0	0.3 (1;1)
*Cyanobacteria*	*Cyanobacteria*		*Family VIII*	*GpVIII*	0.7 (1;1)	0	0.3 (1;1)
			*Family IV*	*GpIV*	0.7 (1;1)	0	0.3 (1;1)
			*Family X*	*GpX*	0.7 (1;1)	0	0.3 (1;1)
			Unclassified		0.7 (1;1)	0	0.3 (1;1)
Total					136;32	175;19	311;47

**FIGURE 1:**
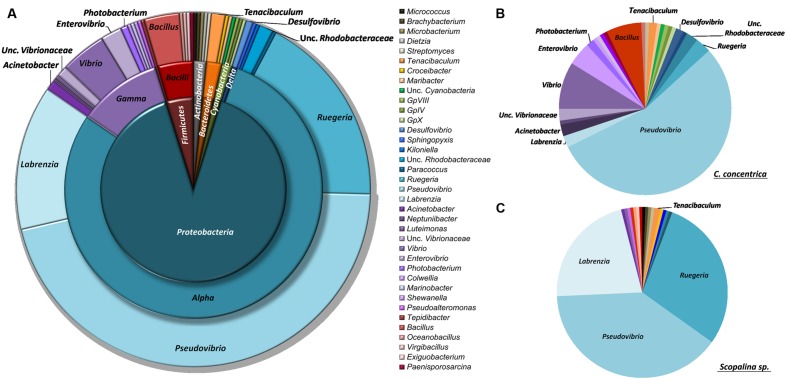
**Taxonomic distribution of sponge-derived bacteria isolated from (A) both sponge species, (B) *Cymbastela concentrica* only and (C) *Scopalina* sp. only, based on RDP classification at 80% confidence threshold**.

Isolated bacteria were overall distributed among five bacterial phyla (**Figure 1**) – *Cyanobacteria* (1.2%), *Actinobacteria* (1.5%), *Bacteroidetes* (1.8%), *Firmicutes* (4.3%), and *Proteobacteria* (90.0%) – with a clear dominance of the latter in both sponge species. Within the *Proteobacteria*, the class *Alphaproteobacteria* was the overall most abundant taxa, making up to 79% of the total abundance of isolated bacteria. However, at the OTU level, *Gammaproteobacteria* showed higher richness, with 13 iOTUs and 11 different bacterial genera, against 10 iOTUs and eight genera within the *Alphaproteobacteria* (**Table 2**). Interestingly, these results differ among sponge species, with *Scopalina* sp. showing higher richness for *Alphaproteobacteria* (six iOTUs) than that found in *Gammaproteobacteria* (three iOTUs).

*Pseudovibrio*, *Ruegeria*, and *Labrenzia* were the most abundant genera for both *C. concentrica* and *Scopalina* sp., making up to 77% of the total abundance of the cultivated fraction. *Vibrio* sp. represents almost 40% of the total gammaproteobacterial abundance and is also present in both sponge species although there is no OTU overlap, meaning that these two different sponge species harbor different *Vibrio* species.

*Bacillus*, phylum *Firmicutes*, is the most diverse bacterial genus found in the present cultivation experiment, with five distinct iOTUs, one of which is shared between the two sponge species.

#### Differences between Sponge Species

Out of the total 47 iOTUs, only four iOTUs were shared among both sponge species, including the three most abundant iOTUs classified as *Pseudovibrio* sp. (*n* = 143), *Ruegeria* sp. (*n* = 55) and *Labrenzia* sp. (*n* = 41), along with one iOTU belonging to genus *Bacillus* sp. (*n* = 2). Although, 77% of the total isolate abundance is shared, both sponge species only have a minor fraction (8.3%) of the total isolate diversity in common. These dissimilarities between the cultivable bacterial communities of *C. concentrica* and *Scopalina* sp. were statistically supported by libshuff analysis (*p* < 0.001).

Bacteria belonging to the family *Rhodobacteraceae* are abundant in the cultured fraction of *Scopalina* sp., with *Pseudovibrio* sp., *Ruegeria* sp., and *Labrenzia* sp. making up to 90% in abundance of the isolates retrieved from this sponge. In *C. concentrica*, while a high abundance of *Alphaproteobacteria* is still observed, there is considerable higher diversity of *Gammaproteobacteria* (10 iOTUs), representing 22% of the isolate total abundance, as opposed to only 2% *Gammaproteobacteria* abundance (corresponding to three iOTUs) observed in *Scopalina* sp. The latter is particularly enriched in *Actinobacteria*, comprising four distinct *Actinomycetales* genera, as opposed to *C. concentrica* from which only one *Streptomyces* sp. isolate was retrieved. In contrast, all four cyanobacterial isolates were recovered only from *C. concentrica*.

#### Influence of Media Composition

**FIGURE 2:**
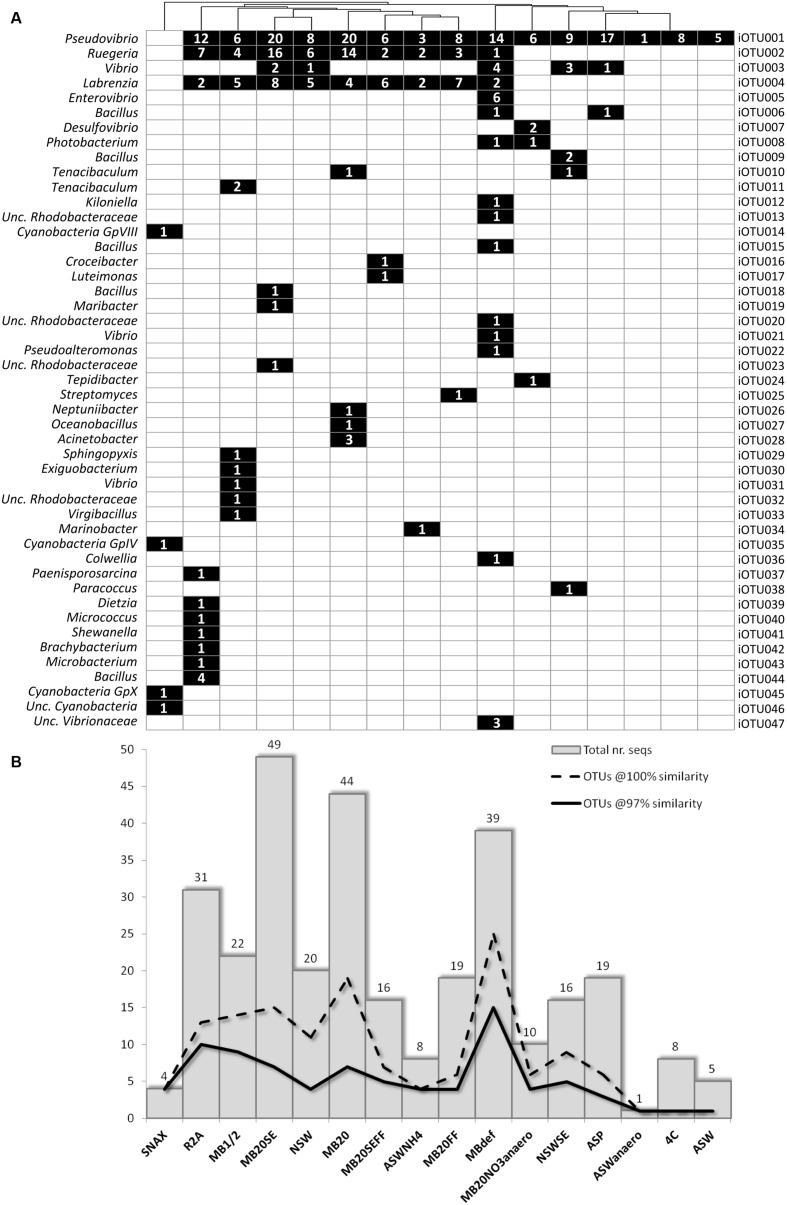
**(A)** Binary map and **(B)** richness and abundance of 16S rRNA gene sequences of bacterial isolates retrieved from each cultivation media; Numbers in squares represent the number of sequences/isolates found in the respective iOTU for that particular isolation medium.

The use of different media and cultivation conditions resulted in a significant increase in richness of the sponge-derived cultivated community, with only seven iOTUs (15% of the total isolate diversity) being common to two or more isolation media (**Figure 2A**).

In general, media with higher nutrient concentration – MB1/2, MBdef, R2A, MB20, and MB20SE – yielded higher bacterial abundances and richness (**Figure 2B**). MB20 and MB20SE media showed the highest bacterial abundances, with 44 and 49 bacterial isolates being retrieved from each media, respectively. However, bacterial 16S rRNA gene sequences clustered into only seven iOTUs (at 97% sequence similarity) for each of these two media. Of these, only the three most abundant iOTUs (iOTU001-*Pseudovibrio* sp., iOTU002-*Ruegeria* sp., and iOTU004-*Labrenzia* sp.) were shared among the two different media (MB20 and MB20SE).

Freezing and thawing the bacterial pellet before plating (MBdef) proved to be an efficient strategy to improve recovery of distinct bacterial genotypes. Thirty nine bacterial isolates clustering into 15 different iOTUs at 97% sequence similarity were retrieved from MBdef, as opposed to only nine iOTU clusters being represented by 22 bacteria isolated from the same medium (MB1/2) without previous freezing/thawing (**Figure 2B**). Again, only the three most abundant iOTUs (iOTU001, iOTU002, and iOTU004) were shared among MB1/2 and MBdef. A closer inspection into the specific MBdef bacterial community revealed an enrichment in *Gammaproteobacteria*, with seven iOTUs (17 isolates) classified as *Gammaproteobacteria*, as opposed to only one gammaproteobacterial isolate found in MB1/2.

The addition of sponge extract (SE) to isolation media allowed the recovery of different bacterial phylotypes when compared to their non-SE counterparts, adding to the overall diversity of the sponge-derived cultivated bacterial community. However, this supplementation did not show to induce enrichment in any particular bacterial phylotype.

Much lower bacterial abundances and richness were observed in floating filter cultures. No microcolonies were observed in any of the floating filters when inspected under a dissection microscope. Also, no macrocolonies were observed in NSWFF and NSWSEFF after 6 months of incubation and the number of isolates derived from MB20FF and MB20SEFF were less than half of their agar based counterparts. Despite their lower abundance, three iOTUs (iOTU0016-*Croceibacter* sp., iOTU0017-*Luteimonas* sp., and iOTU0025-*Streptomyces* sp.) corresponding to three distinct bacterial genera were found exclusively in floating filters media.

Marine broth (MB1/2, MBdef, MB20, MB20SE, MB20FF, and MB20SEFF) and natural seawater (NSW and NSWSE) based media were generally non-specific, with isolates coming from these media collectively spanning across all bacterial phyla found in this study, except *Cyanobacteria*. Very low-nutrient media (ASW and 4C) did not recover any specific bacteria, even when incubated in anaerobic conditions (ASWanaero), with all isolates retrieved from these media classified as *Pseudovibrio* sp. (iOTU001). ASP, a medium formulated to mimic the sponge environment by incorporating components of the sponge matrix (silica and spongin) and the addition of sponge extract, also failed in the recovery of specific bacteria, with all isolates also being found in other media. Media that targeted microorganisms involved in nitrogen cycling (ASWNH4 and MB20NO3anaero, for nitrifying and denitrifying organisms, respectively) yielded isolates that could not be found in any other media, with members of genera *Photobacterium* and *Desulfovibrio* previously known to be capable of nitrate reduction (Keith and Herbert, 1983; Thompson et al., 2005). SNAX medium efficiently selected targeted organisms, with four different cyanobacterial isolates being retrieved from *C. concentrica*. Interestingly, all isolates retrieved from anaerobic media (ASWanaero and MB20NO3anaero) come from *C. concentrica*, although anaerobic functions have also been previously detected in *Scopalina* sp. (Fan et al., 2012b).

### Cultivation Independent Analysis of the Sponge Community

After quality-filtering, removing of rare sequences and correction for gene copy number estimates, 1,321,731 sequences for the v4 region of the 16S rRNA gene were obtained from 27 samples, which clustered into 4566 uOTUs at 97% similarity (Supplementary Table S1). After sub-sampling to the size of the smallest sample (Scop3SP, 19287 sequences), observed richness was shown to be around half of that estimated by the Chao1 index (Supplementary Figure S1), meaning that rare uOTUs make up around half of the community data. No statistically significant differences (ANOVA two-way) for richness and diversity estimates were found between samples that could be correlated to date of collection, sponge species or sample processing.

**FIGURE 3:**
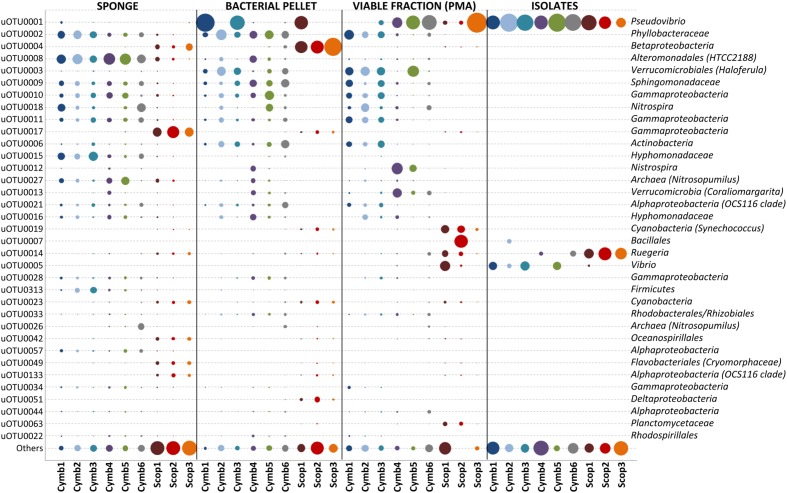
**Relative abundances of the 35 overall most abundant uOTUs (normalized for gene copy number)**. Relative abundances shown in proportion to area of bubbles; uOTU taxonomy shown up to the lowest taxon based on SILVA, RDP and Greengenes classification, with a confidence threshold >60%.

Around 30% of the total diversity detected in SP samples were common to both sponge species, representing approximately 98% of the total abundance in these samples. Although, these common uOTUs could be found in both sponge species, their abundance profiles are quite disparate: abundant uOTUs in *C. concentrica* are practically absent in *Scopalina* sp. and vice-versa (**Figure 3**). The 35 overall most abundant uOTUs represented up to 84% in abundance of the total cultivation-independent bacterial community (**Figure 3**). The bacterial composition was consistent among replicates of the same sponge species, regardless of the different collection dates (*C. concentrica* only), but differs significantly for each sponge species, with uOTUs belonging to *Alteromonadales* (uOTU008), *Phyllobacteraceae* (uOTU002), and *Hyphomonadaceae* (uOTU015) being the most abundant in *C. concentrica* (average relative abundances 23, 9, and 9%, respectively), and *Scopalina* sp. being particularly rich in uOTUs assigned to the *Gammaproteobacteria* (uOTU017) and *Betaproteobacteria* (uOTU004), with average relative abundances of 24 and 7%, respectively.

**FIGURE 4:**
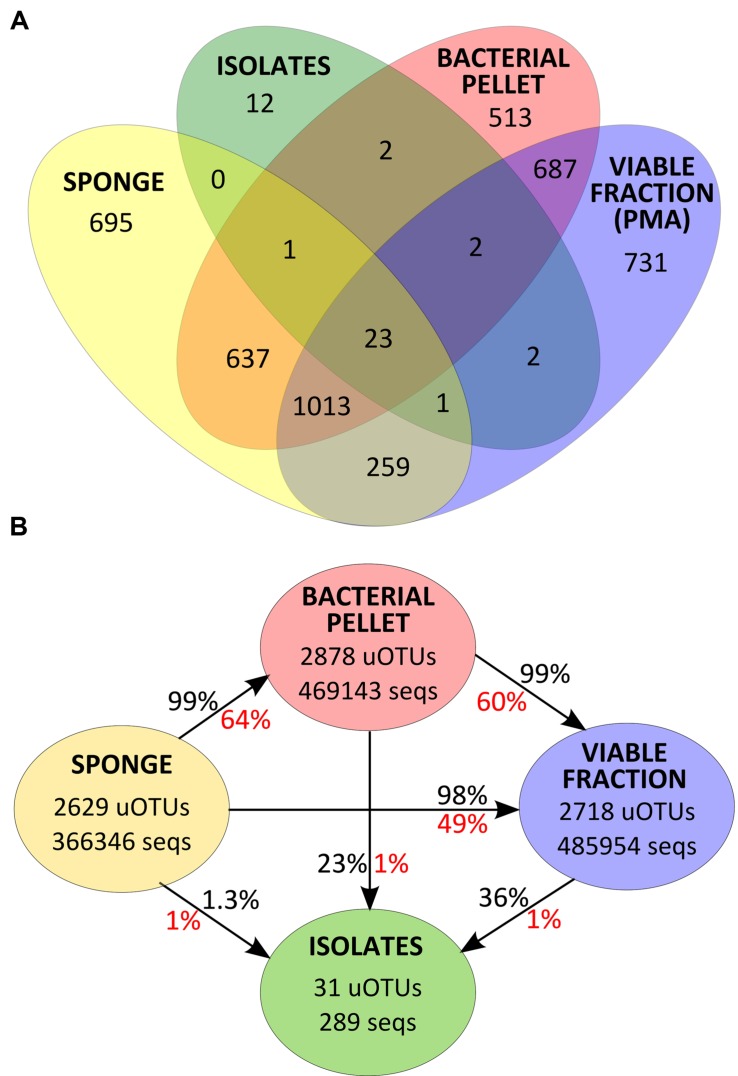
**(A)** Venn diagram for uOTU distribution of v4-16S rRNA gene sequences at 97% similarity for each sample group. **(B)** Total number of v4-16S rRNA gene sequences and uOTUs at 97% similarity in each sample group and percentage of diversity (in red) and abundance (in black) recovered after each sample processing step.

Despite an occasional enrichment in uOTU001 (*Pseudovibrio* sp.), the composition of the bacterial pellet broadly reflects that of the initial sponge sample, although for some particular uOTUs (e.g., uOTU017, belonging to *Gammaproteobacteria*, and uOTU004, belonging to *Betaproteobacteria* in *Scopalina* sp.), abundance and/or viability was lost or considerably reduced on sample processing (**Figure 3**). While abundant members of the sponges’ bacterial communities were not greatly affected by sample processing, 36% of the initial diversity was lost in the microbial enrichment step, 51% of this initial diversity lost viability and only 1% was recovered in cultivation (**Figure 4**). These ‘lost’ OTUs represent low abundance bacteria that, although they comprehend almost half of the community diversity, comprise only 1–2% of the total abundance. Still, the largest shift in the community profile occurs in the cultivation step: the most abundant bacteria in the initial sponge sample were not obtained in cultivation whereas taxa originally not predominant in the sponge seem to have been enriched (**Figure 3**).

### Overlap of the Community Composition and Cultured Isolates

Thirty one of the 43 uOTUs found in the cultivated fraction were shared with the cultivation-independent community assessment (**Figure 4A**). These common 31 uOTUs make up a total of 289,453 sequences (corrected for estimated gene copy number), representing an overlap of 22% with the total community abundance (SP, BAC, and PMA samples, collectively). Around 97% of the total isolates’ abundance (279 sequences distributed among 25 uOTUs) could be detected in the sponge tissue (SP samples). Another six uOTUs, representing 10 isolates, could not be traced back to the sponge but were found in the bacterial pellet and/or in the viable fraction (**Figure 4A**). The latter sequences, along with the remaining 12 uOTUs (corresponding to 12 individual sequences) found exclusively in the isolates, could either be derived from contaminant or very low abundance bacteria that could not be detected using molecular methods.

**FIGURE 5:**
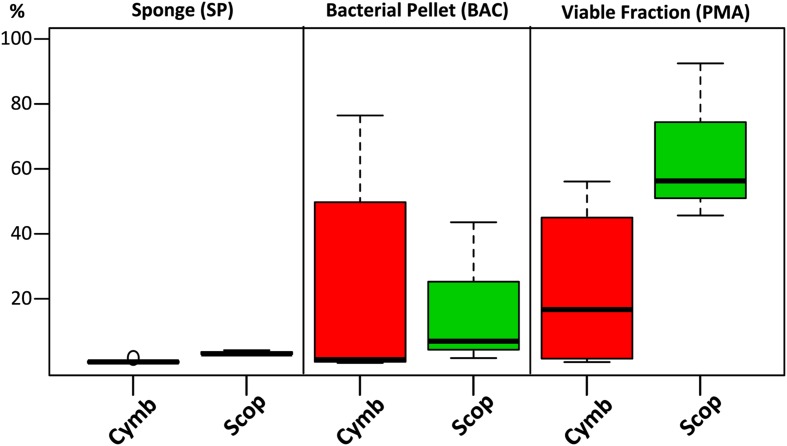
**Combined relative abundances (corrected for gene copy number estimates) of uOTUs recovered in the cultured fraction after each sample processing step**. For each sponge specimen and sample treatment, relative abundances of uOTUs found in common with the cultured fraction were combined and plotted by grouping them according to sponge species.

**FIGURE 6:**
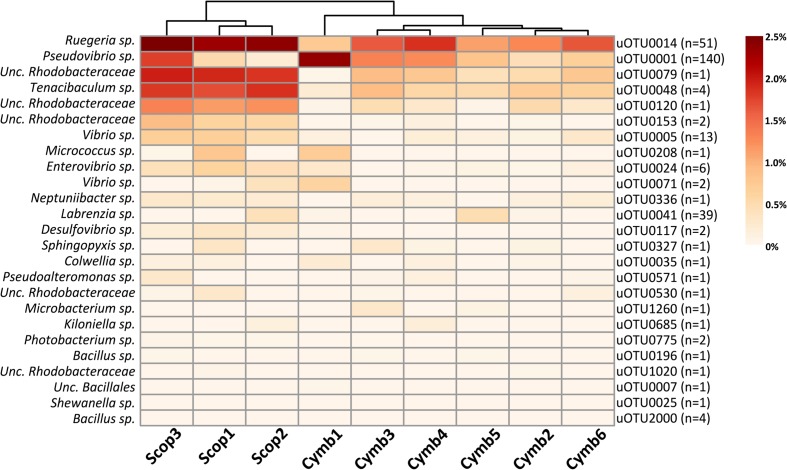
**Heat map of relative abundances (corrected for gene copy number estimates) of uOTUs originally found in the sponge overlapping the cultured fraction**. *n* stands for the number of isolate sequences found to cluster within that specific uOTU.

Although the cultured fraction overlaps only a small percentage of the original sponge-associated bacterial abundance (0.2–1.8% for *C. concentrica* and 2.7–4.2% for *Scopalina* sp.), it represents 23% of the total abundance found in the microbial cell pellets (BAC samples) and 36% of the total abundance of cells that remained viable after sample processing (PMA samples; **Figure 4B**). These results are, however, highly variable between different sponge species, with isolates representing 0.5–56% and 46–92% of the viable bacteria associated with *C. concentrica* and *Scopalina* sp., respectively (**Figure 5**). The isolates found to be most abundant in sponge samples were classified as *Ruegeria* sp. (0.04–2.23% abundance range in SP samples), followed by *Pseudovibrio* sp. (0.006–1.65% abundance range in SP samples), an unclassified *Rhodobacteraceae* (0.001–0.70% abundance range in SP samples) and *Tenacibaculum* sp. (0.006–0.56% abundance range in SP samples; **Figure 6**). *Labrenzia* sp. (uOTU0041), although being one of the most abundant isolates and the fifth most abundant uOTU in PMA samples, was not found to be predominant in the sponge, representing only 0.0012–0.017% of the total abundance in SP samples.

## Discussion

The bacterial communities associated to *C. concentrica* and *Scopalina* sp. are very similar between different individuals and highly specific to their hosts (**Figure 3**). The similarity of the uOTU abundance profiles in multiple *C. concentrica* samples taken from the field 5 months apart shows that its microbial community remained stable through different seasons. This observation confirms previous works that used denaturing gradient gel electrophoresis (DGGE) banding patterns to show that the bacterial community composition of *C. concentrica* remained stable across time and space (Taylor et al., 2005). Abundant bacteria found in the present study, both in the sponge and in the bacterial pellet – *Betaproteobacteria*, *Alphaproteobacteria* (OCS116 clade) and *Deltaproteobacteria* for *Scopalina* sp. and *Phyllobacteraceae*, *Hyphomonadaceae*, *Nitrosomonadaceae*, *Thaumarchaeota*, *Sphingomonadaceae*, and *Actinobacteria* for *C. concentrica* – have also been found to be prominent in previous studies (Taylor et al., 2004; Thomas et al., 2010; Fan et al., 2012a,b), showing that, in a broader time scale, the bacterial community profiles of these sponge species remained stable, at least regarding their most abundant members.

We were able to identify two ecological types of bacteria: ‘specialists’ that were only present in one sponge species and ‘non-specialists’ that were present in both sponge species. In the latter category are included ‘sponge associates’ – bacteria found in multiple sponge hosts but not in seawater – and ‘generalists’ – bacteria found in sponges and seawater (Taylor et al., 2004) – but this distinction was not possible in the present study as no seawater samples were analyzed. The ‘specialists’ – uOTUs exclusive to each sponge species – account for diversity estimates of 61 and 48% in *C. concentrica* and *Scopalina* sp., respectively. Considering that rare uOTUs (*n* ≤ 2), possibly specialists, were removed during sequence processing, the bacterial communities of *C. concentrica* and *Scopalina* sp. are highly specific to each sponge host. Cultivated communities also reflected this host specificity, with isolates retrieved from different sponge species showing an overlap of only ∼8% in diversity.

With the exception of *Betaproteobacteria* (uOTU0004) and *Gammaproteobacteria* (uOTU0017) found in *Scopalina* sp. (discussed below), cell fractionation did not have a major impact on the most abundant members of the sponge bacterial communities. A total of 1036 uOTUs were shared between SP, BAC and PMA samples, representing around 98% of the total abundance of v4-16S rRNA gene sequences of bacteria that were enriched in the microbial pellet and maintained viability. However, sample processing had a high impact on low abundance bacteria, with considerable loss of diversity, where 36% of the low abundance uOTUs found in the sponge were not recovered in the microbial pellet and 51% could not be detected in PMA samples.

The density of the collagenous matrix of sponges has been pointed out as a discriminating factor for different bacterial recovery rates in methodologies that rely on sample processing, due to variability in mechanical ease of bacterial cell detachment and disruption from the sponge matrix (Hardoim et al., 2014). *C. concentrica* has a tough leathery consistency that makes it more difficult to homogenize in comparison to *Scopalina* sp. Although, no major differences were found in the recovery rates observed for each sponge species, inefficient disruption of the sponge matrix and differential bacterial detachment, along with inherent losses in the centrifugation and filtration steps of the microbial enrichment methodology, constitute factors that may have contributed to the loss of diversity in the microbial pellet.

Most of the isolates could be detected in the original sponge samples, representing 0.2–4.2% of the sponges’ total microbial abundance. Previous cultivability estimates – defined by the number of colony forming units (CFUs) divided by the total number of bacteria present in the sponge (Santavy et al., 1990; Friedrich et al., 2001; Webster et al., 2001) or by the number of different bacterial species that could be cultured divided by the total bacterial diversity determined by DGGE (Olson and McCarthy, 2005; Sipkema et al., 2011) – ranged from 0.1 to 14%, for different sponge species. However, the methodologies used by these surveys to determine total bacterial counts and/or diversity disregard several aspects that might severely impact cultivability: (1) the ratio CFU/microscopy cell counts assumes even relative abundances between all microbial species or phylotypes that constitute the community; (2) with the exception of Hardoim et al. (2014), no other study contemplated the influence of sample processing in recovering the original bacterial diversity in the sponge; (3) cell viability, evaluated not on total active cell counts but on how the chosen experimental conditions can impact the integrity of different bacterial phylotypes, was never previously addressed by any other cultivation based study, to the best of our knowledge.

The loss of two dominant bacterial groups in *Scopalina* sp. (uOTU0004 and uOTU0017) clearly demonstrates the effects of sample processing and viability on bacterial recovery (**Figure 3**). While uOTU0004 was enriched in the microbial pellet, its viability was greatly compromised. With respect to uOTU0017, sample processing had a greater impact, considerably reducing its abundance in the bacterial pellet and an even smaller fraction remained viable. Upon sample processing, potentially damaging conditions such as the presence of oxygen, variable osmotic pressure, different pH, changes in salinity and mechanical shearing can cause cell disruption and affect viability. The ability of the sample processing methodology to yield bacteria that remain viable will ultimately dictate their amenability to cultivation. In the present work, isolates represented a high and largely variable proportion of the total viable fraction abundance (**Figure 5**), showing that culturing captured a few of the mostly viable groups of bacteria – like *Pseudovibrio* sp., *Ruegeria* sp., *Labrenzia* sp., and *Vibrio* sp. – although these would not be abundant in the original sponge samples. Cultivation has often been thought to select for opportunistic ‘weedy’ bacteria that, although not abundant, grow quickly in nutrient-rich media and out-compete other slow growing species (Garland et al., 2001). The most abundant bacteria obtained in culture were also the most abundant in the viable fraction. This observation shows that cultivation might, to some extent, actually reflect the relative abundances of the viable bacterial community rather than the sponge community distribution estimated by direct molecular approaches. These results highlight the need to perform preliminary experiments to assess the ability of each sample processing method to accurately estimate the actual *in situ* sponge bacterial community and yield viable cultivable cells, as these may differ with methodology, bacterial composition and sponge species.

In this work, most of the isolated bacteria (97% abundance) were detected in the original sponge community (SP samples) with variable relative abundances, representing an improvement in comparison to previous studies. Li et al. (2011) used complementary cultivation-dependent and cultivation-independent methods based on PCR-restriction fragment length polymorphism (PCR-RFLP) and 16S rRNA gene libraries and found no overlap between the cultured and uncultured communities of the sponge *Gelliodes carnosa*. Sipkema et al. (2011) performed an extensive cultivation experiment using *Haliclona* sp., yielding 3900 isolates clustered into 205 OTUs, of which only 17 OTUs could be identified in a previously generated clone library. More recently, Montalvo et al. (2014) analyzed the bacterial diversity of *Xestospongia* sp. sponges using pyrosequencing and cultivation and only one OTU was found in common. These disparities probably relate to the use of different molecular approaches, with the Illumina sequencing technology used in the present study providing a deeper characterization of the sponges’ microbiomes. Also, different molecular methods and experimental steps can potentially produce biases/artifacts that significantly influence biological interpretations of the dataset (Fan et al., 2012a; Ushio et al., 2014).

*Pseudovibrio* sp., *Ruegeria* sp., and *Labrenzia* sp. were the most commonly isolated bacteria in the present study. Although they were not prevalent members of the original sponge community, with a combined relative abundance of 0.8% in SP samples, they represent 32% of the total viable fraction abundance. These members of the *Rhodobacteraceae* family seem to be abundant and widespread in the marine environment and have been commonly isolated from sponges and other organisms and also seawater (Biebl et al., 2007; Brinkhoff et al., 2008; Menezes et al., 2010). Their diverse and versatile metabolic features have been reported previously (Brinkhoff et al., 2008; Bondarev et al., 2013) and were once again highlighted in the present study by their ability to grow in a wide range of media compositions and incubation conditions. In addition to these widespread and ubiquitous bacteria, a multitude of other media-specific and less abundant isolates resulted from a broad range of non-traditional culturing techniques, coupled with long term incubations (6 months) and low colony densities, used in this work. This observation, along with previous findings (Sipkema et al., 2011; Lavy et al., 2014) emphasize that a variety of methods ought to be incorporated in culturing experiments to address the different metabolic needs of sponge associated bacteria. Despite the extensive culturing effort applied here, there is still a substantial gap between the bacterial community residing in the sponge and what was recovered in culture. Many bacteria, albeit remaining viable, did not thrive in the culturing conditions provided. Cell-to-cell interactions, either through shared metabolic pathways, chemical communication or by direct physical contact, local or temporal variations within the host, such as compartmentalization (Fieseler et al., 2004) or transient anaerobic patches (Hoffmann et al., 2005), can create diverse micro-niches to which sponge symbionts are likely to be exposed. The failure to mimic these growth conditions *in vitro*, along with the loss of viability, likely constitutes the main factors for non-cultivability.

## Conclusion

Earlier studies estimated that only as much as 1% of the total bacteria found in a variety of aquatic habitats were amenable to cultivation, with the remaining 99% constituting the “vast unculturable majority” (Rappé and Giovannoni, 2003). Two main constraints were hypothesized to account for these low recoverability rates: (1) that bacteria could not be viable, and (2) that the growth conditions were not adequate. Several authors dwelled on this matter, finding that most bacteria in aquatic habitats were in fact metabolically active albeit not being recovered in plating procedures (Staley and Konopka, 1985). However, this evidence was based on microscopy counts, completely disregarding bacterial diversity and unevenness of community relative abundances. Since then, several efforts have been made in trying to overcome this 1% boundary, with reports of up to 14% bacterial recoverability in the marine sponge *Haliclona* sp. (Sipkema et al., 2011). Still, the major bacterial groups found in sponges remain recalcitrant to cultivation (Hardoim et al., 2014; Montalvo et al., 2014).

In order to identify the constraints that are responsible for the low recoverability of sponge bacteria cultivation, we used a combination of culture-dependent and independent community analysis to determine the impact of sample processing and growth conditions on the success of cultivation. We found that sponge bacteria can be divided into four groups in regards to susceptibility to the sample processing method and amenability to cultivation: (1) bacteria that were not recovered by the fractionation process to obtain a microbial pellet; (2) bacteria that were present in the microbial pellet but whose viability was compromised after sample processing and hence could not grow in culture; (3) bacteria in the pellet that, although maintaining viability, could not be cultured; and (4) bacteria that remained viable and were recovered in culture. While low abundances, inefficient tissue disruption, differential bacterial detachment and exposure to damaging conditions during sample processing can account for groups 1 and 2, group 3 constitutes the major proportion of bacteria and is most likely explained by unsuitability of the growth conditions provided. Isolated bacteria (group 4), although they are members of the sponge ‘rare biosphere,’ can represent a significant proportion of the bacterial viable fraction. Cultivation is therefore shaped not only by the growth conditions provided, but also by the different cell viabilities of the bacteria that constitute the microbial inoculum.

The continuously expanding knowledge about the sponge holobiont – its physiology, metabolism and the specificities of its microbial associations – will be crucial in broadening our understanding and ability to isolate these microbes that, despite extensive culturing efforts, still elude cultivation.

## Author Contributions

AE, NA, and MN performed the laboratory experiments. AE and TT conceived and designed the study, analyzed the data, compiled the results and wrote the manuscript draft. All authors revised the draft, approved the final manuscript version and are accountable for all aspects of the work.

## Conflict of Interest Statement

The authors declare that the research was conducted in the absence of any commercial or financial relationships that could be construed as a potential conflict of interest.

## Footnotes

1 http://www.ebi.ac.uk/ena

2 http://www.ncbi.nlm.nih.gov/sra
