# Supplementary material for: Sample Processing Impacts the Viability and Cultivability of the Sponge Microbiome
Source: Front Microbiol. 2016 Apr 12;7:499. doi: 10.3389/fmicb.2016.00499 (PMC4876369; doi:10.3389/fmicb.2016.00499)
Supplement: Supplementary file 1 [file Data_Sheet_1.DOCX]

***Supplementary Material***

**Sample processing impacts the viability and cultivability of the sponge microbiome**

**Ana I. S. Esteves, Nimra Amer, Mary Nguyen, Torsten Thomas^*^**

*** Correspondence:** Torsten Thomas: t.thomas@unsw.edu.au

**1. Supplementary Figures and Tables**

**Supplementary Table 1 -** Sample information for v4-16S rRNA gene sequencing processing; na stands for not applicable.

| **Sample** | **Initial nr seqs** | **Seqs after quality filtering** | **Unique seqs** | **OTUs 97% before doubleton filtering** | **Seqs after doubleton filtering** | **OTUs 97% after doubleton filtering** | **Seqs after gene copy nr correction** |
| --- | --- | --- | --- | --- | --- | --- | --- |
| **Cymb1SP** | 105643 | 82889 | 4612 | 1588 | 81799 | 535 | 51546 |
| **Cymb2SP** | 118238 | 82194 | 4263 | 1644 | 81200 | 708 | 46149 |
| **Cymb3SP** | 99264 | 68486 | 4287 | 1794 | 67369 | 747 | 35972 |
| **Cymb4SP** | 97664 | 76459 | 4548 | 1860 | 75360 | 833 | 44150 |
| **Cymb5SP** | 99011 | 79355 | 4502 | 1837 | 78189 | 730 | 50482 |
| **Cymb6SP** | 102707 | 82090 | 4798 | 2035 | 80820 | 863 | 53818 |
| **Scop1SP** | 90387 | 72978 | 5721 | 2459 | 71407 | 1092 | 37846 |
| **Scop2SP** | 62500 | 51307 | 3808 | 1668 | 50340 | 803 | 27096 |
| **Scop3SP** | 47849 | 38826 | 3365 | 1440 | 38026 | 732 | 19287 |
| **Cymb1BAC** | 137037 | 119893 | 8309 | 1372 | 118845 | 348 | 59392 |
| **Cymb2BAC** | 116642 | 98324 | 6047 | 2194 | 96869 | 819 | 49789 |
| **Cymb3BAC** | 150104 | 127358 | 9139 | 2219 | 125776 | 731 | 62381 |
| **Cymb4BAC** | 117975 | 95248 | 4889 | 1733 | 94112 | 652 | 53315 |
| **Cymb5BAC** | 104696 | 86198 | 5603 | 2037 | 84756 | 635 | 47804 |
| **Cymb6BAC** | 113886 | 92533 | 6514 | 2498 | 90908 | 952 | 48187 |
| **Scop1BAC** | 138590 | 127217 | 8537 | 2437 | 125550 | 878 | 61381 |
| **Scop2BAC** | 93417 | 83208 | 5109 | 2006 | 81976 | 862 | 41062 |
| **Scop3BAC** | 107900 | 99377 | 4925 | 1933 | 98150 | 795 | 45831 |
| **Cymb1PMA** | 117202 | 101050 | 5492 | 1592 | 99879 | 460 | 49549 |
| **Cymb2PMA** | 133233 | 112443 | 5866 | 2136 | 111047 | 804 | 66504 |
| **Cymb3PMA** | 116935 | 98270 | 6195 | 2227 | 96760 | 824 | 51124 |
| **Cymb4PMA** | 109834 | 102811 | 5462 | 1492 | 101874 | 582 | 66337 |
| **Cymb5PMA** | 119712 | 113592 | 5937 | 1533 | 112577 | 559 | 75984 |
| **Cymb6PMA** | 115348 | 108761 | 8023 | 2235 | 107373 | 1001 | 56144 |
| **Scop1PMA** | 139679 | 133650 | 6106 | 1431 | 132620 | 419 | 30462 |
| **Scop2PMA** | 120661 | 114993 | 6241 | 1389 | 114062 | 478 | 24481 |
| **Scop3PMA** | 136515 | 131410 | 8746 | 1264 | 130571 | 451 | 65368 |
| **Total Illumina** | 3012303 | 2580920 | 146726 | 34294 | 2548215 | 4566 | 1321442 |
| **Cymb1isol** | 25 | 20 | 10 | 8 | 18 | 6 | na |
| **Cymb2isol** | 23 | 20 | 7 | 6 | 19 | 5 | na |
| **Cymb3isol** | 31 | 29 | 7 | 5 | 29 | 5 | na |
| **Cymb4isol** | 31 | 28 | 12 | 12 | 25 | 9 | na |
| **Cymb5isol** | 14 | 13 | 4 | 4 | 12 | 3 | na |
| **Cymb6isol** | 25 | 24 | 9 | 8 | 22 | 6 | na |
| **Scop1isol** | 77 | 71 | 12 | 10 | 70 | 9 | na |
| **Scop2isol** | 47 | 46 | 8 | 5 | 46 | 5 | na |
| **Scop3isol** | 53 | 50 | 10 | 10 | 48 | 8 | na |
| **Total isolates** | 326 | 301 | 79 | 43 | 289 | 31 | na |

**Supplementary Figure 1 -** Analysis of bacterial richness and diversity in marine sponge samples after correction for gene copy number estimates and sub-sampling to size of smallest sample (Scop3SP, 19287 sequences). **(A)** Observed and estimated (Chao1) richness; **(B)** Shannon diversity indexes. OTUs were determined at 97% sequence similarity. Values on bars are mean standard deviation of three replicates within each sample category.


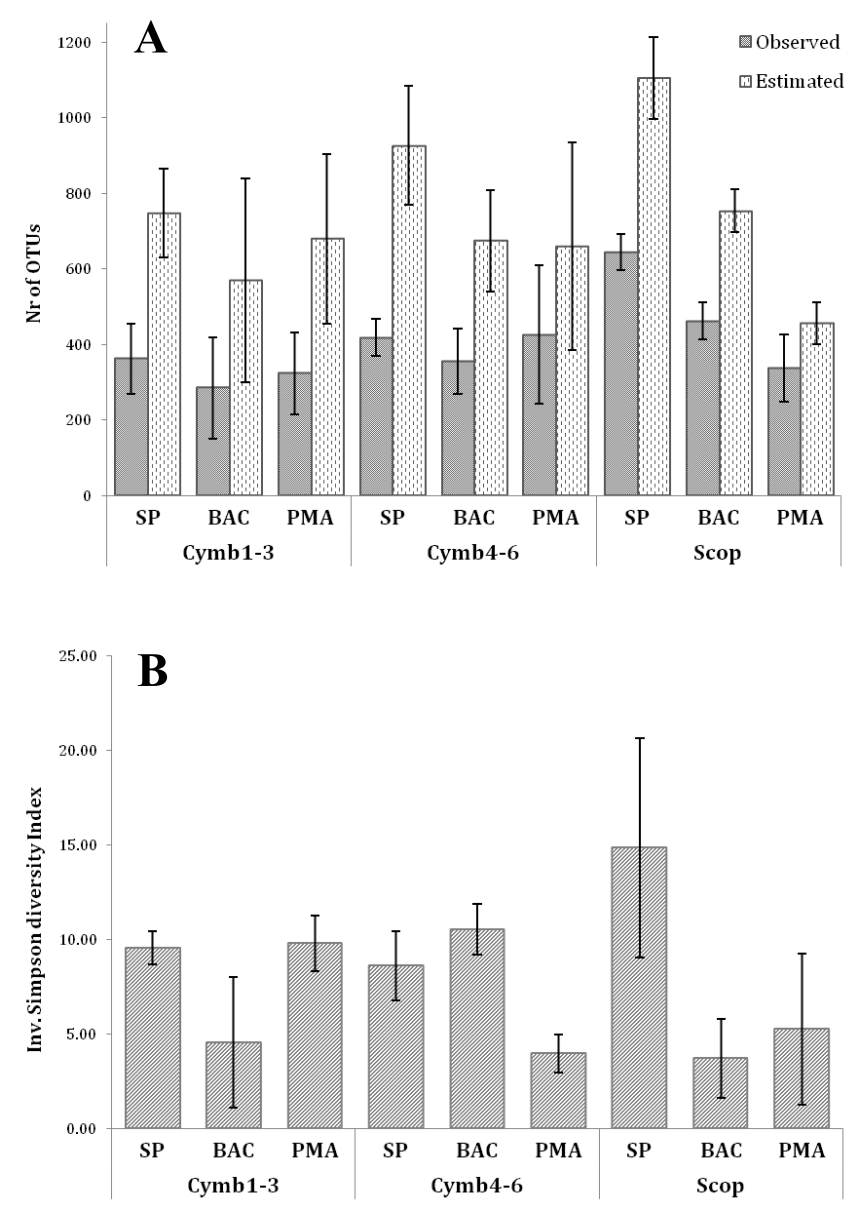


**Supplementary Figure 2 -** Optimal 16S rRNA gene maximum-likelihood (ML) tree for representative sequences of iOTUs (in bold red) and uOTUs (in bold black) found in the cultured fraction. In brackets, n stands for the number of isolates from this study, if more than one could be represented by one single tree leaf. ML bootstrap values (> 75%) and bayesian posterior probabilities (> 0.95) are shown above and below branches, respectively. The tree is rooted with the phylum *Thermodesulfobacteria*.
