# Supplementary figures and images for: Sample Processing Impacts the Viability and Cultivability of the Sponge Microbiome
Source: Front Microbiol. 2016 Apr 12;7:499. doi: 10.3389/fmicb.2016.00499 (PMC4876369; doi:10.3389/fmicb.2016.00499)

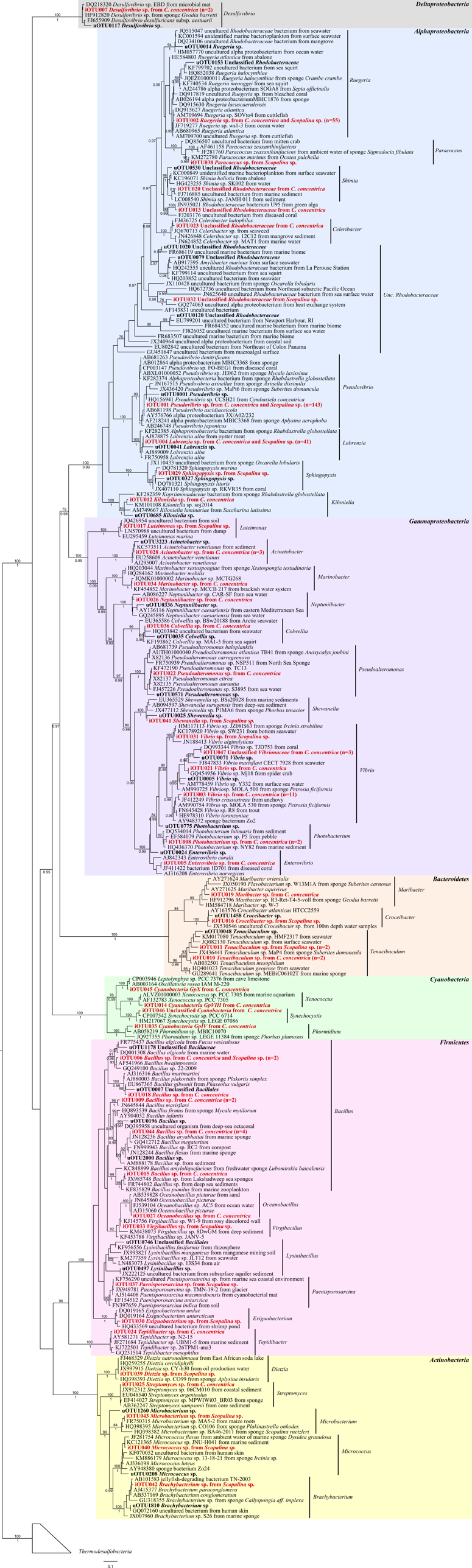

Supplement: Supplementary file 2 [file Image_1.TIFF]
